# High-Quality Genome Sequence of *Xanthomonas axonopodis* pv. glycines Strain 12609 Isolated in Taiwan

**DOI:** 10.1128/genomeA.01695-16

**Published:** 2017-02-23

**Authors:** Shu-Fen Weng, An-Chi Luo, Che-Jui Lin, Tsai-Tien Tseng

**Affiliations:** a Institute of Molecular Biology, National Chung Hsing University, Taichung, Taiwan; b Department of Molecular and Cellular Biology, Kennesaw State University, Kennesaw, Georgia, USA

## Abstract

The genomic sequence was determined for *Xanthomonas axonopodis* pv. glycines strain 12609, isolated in Taiwan. Based on the genome sequence, we predicted the encoded genes, rRNA, tRNA, a plasmid sequence, secretion systems, cyclic GMP- and cyclic di-GMP-mediated pathways, and the gene cluster* rpfABCHGDE* (regulation of pathogenicity factor).

## GENOME ANNOUNCEMENT

The genus *Xanthomonas* contains 27 closely related plant-pathogenic species (1). *Xanthomonas axonopodis* pv. glycines is the bacterium causing pustules in soybeans, causing tremendous losses (2). It exhibits rapid cell death (RCD) response in rich media, in which the intracellular cyclic AMP (cAMP) levels are high until the onset of stationary phase but decline rapidly afterward (3). These features render *Xanthomonas axonopodis* pv. glycines suitable for comparative study with other xanthomonads, e.g., *Xanthomonas campestris* pv. campestris, which infects crucifers, does not exhibit RCD, and has no detectable levels of cAMP (4), although the adenylate cyclase (Cya) predicted for *X. axonopodis* pv. glycines 12609 (Cya_*Xag12609*_) shares 92% identity with the Cya of *X. campestris* pv. campestris strain 17 (Cya_*Xcc17*_) responsible for cAMP synthesis (5). For future study, the genome sequence of *X. axonopodis* pv. glycines 12609 (also named BCRC 12609) was determined by next-generation sequencing. Twenty-eight contigs were obtained from 30-fold coverage, giving 5,183,780 nucleotides (nt) (G+C content, 65%). Annotation with best-placed reference protein set and GeneMarkS+ version 3.3 (6) predicted 4,475 genes, 4,220 proteins, four rRNA genes, 52 tRNA genes, and a DNA sequence 99% identical to the plasmid unnamed2 (5,753 bp) in *X. axonopodis* pv. glycines CFBP2526 (7) and 100% identical to the 6-kb chromosomal copy of *X. axonopodis* pv. glycines strain 8ra (8).

Cyclic GMP (cGMP)- and cyclic di-GMP (c-di-GMP)-mediated pathways regulate multiple functions, including pathogenesis in xanthomonads (9). These pathways at least involve (i) diguanylate cyclase (DGC) and guanylate cyclase (GCase) required for the synthesis of c-di-GMP and cGMP, respectively; and (ii) cAMP receptor protein-like protein (Clp), the transcription factor required for the expression of hundreds of genes in *X. campestris* pv. campestris (10). In the presence of c-di-GMP, the Clp-promoter binding activity* in vitro* is lowered (11). Homologs of these proteins are found and are highly conserved in *X. axonopodis* pv. glycines 12609, suggesting that cGMP- and c-di-GMP-mediated pathways are operative in *X. axonopodis* pv. glycines 12609.

Protein secretion plays a central role in modulating the interactions of bacteria with their environments, including pathogenesis; six classes are known in Gram-negative bacteria (12). All these systems are present in *X. axonopodis* pv. glycines; in contrast, *X. campestris* pv. campestris has no type VI secretion system (T6SS). Whether T6SS is involved in functions other than pathogenesis, e.g., RCD and host specificity determination, deserves further study.

The gene cluster* rpfABCHGDE* (regulation of pathogenicity factor) is involved in the synthesis of diffusible signal factor, which is required for the production of virulence factors (13). *X. axonopodis* pv. glycines 12609 also contained the *rpf* cluster but not *rpfI*, whose homolog in *X. campestris* pv. campestris encodes a regulatory protein for the production of protease and endoglucanase (14). However, our plate assay indicated that the levels of these enzymes in *X. axonopodis* pv. glycines 12609 were as high as those in the virulent *X. campestris* pv. campestris strain 17 (15), suggesting that their production in *X. axonopodis* pv. glycines is regulated differently.

Filamentous phage phiLf of *X. campestris* pv. campestris can integrate into the host chromosome by site-specific integration using the *dif* (deletion induced filamentation of chromosome) sequence as the *attB* site (16). Prophages similar to phiLf are present in the genomes of *X. axonopodis* pv. glycines strain 8ra (8) and *X. campestris* pv. vesicatoria strain 85-10 (17) but not in that of *X. axonopodis* pv. glycines 12609.

### Accession number(s).

The draft genome sequence of *X. axonopodis* pv. glycines 12609 (Bioresource Collection and Research Center, Taiwan) is now available in the GenBank database under accession number MKCQ00000000.
